# Altered Esophageal Mucosal Structure in Patients with Celiac Disease

**DOI:** 10.1155/2016/1980686

**Published:** 2016-02-29

**Authors:** María Inés Pinto-Sánchez, Fabio D. Nachman, Claudia Fuxman, Guido Iantorno, Hui Jer Hwang, Andrés Ditaranto, Florencia Costa, Gabriela Longarini, Xuan Yu Wang, Xianxi Huang, Horacio Vázquez, María L. Moreno, Sonia Niveloni, Premysl Bercik, Edgardo Smecuol, Roberto Mazure, Claudio Bilder, Eduardo C. Mauriño, Elena F. Verdu, Julio C. Bai

**Affiliations:** ^1^Farncombe Family Digestive Health Research Institute, McMaster University, Hamilton, ON, Canada L8S 4K1; ^2^Department of Medicine, “Dr. Carlos Bonorino Udaondo” Gastroenterology Hospital, 1264 Buenos Aires, Argentina; ^3^Favaloro University Hospital, 1093 Buenos Aires, Argentina; ^4^Consejo de Investigación en Salud, MSAL, Gobierno de la Ciudad Autónoma de Buenos Aires, 1425 Buenos Aires, Argentina; ^5^Gastroenterology Chair, Universidad del Salvador, 1056 Buenos Aires, Argentina

## Abstract

*Background/Aim*. Reflux symptoms (RS) are common in patients with celiac disease (CD), a chronic enteropathy that affects primarily the small intestine. We evaluated mucosal integrity and motility of the lower esophagus as mechanisms contributing to RS generation in patients with CD.* Methods*. We enrolled newly diagnosed CD patients with and without RS, nonceliac patients with classical reflux disease (GERD), and controls (without RS). Endoscopic biopsies from the distal esophagus were assessed for dilated intercellular space (DIS) by light microscopy and electron microscopy. Tight junction (TJ) mRNA proteins expression for zonula occludens-1 (ZO-1) and claudin-2 and claudin-3 (CLDN-2; CLDN-3) was determined using qRT-PCR.* Results*. DIS scores were higher in patients with active CD than in controls, but similar to GERD patients. The altered DIS was found even in CD patients without RS and normalized after one year of a gluten-free diet. CD patients with and without RS had lower expression of ZO-1 than controls. The expression of CLDN-2 and CLDN-3 was similar in CD and GERD patients.* Conclusions*. Our study shows that patients with active CD have altered esophageal mucosal integrity, independently of the presence of RS. The altered expression of ZO-1 may underlie loss of TJ integrity in the esophageal mucosa and may contribute to RS generation.

## 1. Introduction

Celiac disease (CD), a gluten-dependent enteropathy occurring in genetically predisposed subjects, is a common condition with an estimated prevalence of 1% in the general population [1, 2]. The disorder is initiated by the interaction between proteolytic-resistant gluten peptides and the small intestinal mucosa [3]. These mucosal events involve innate immune activation and disruption of the intestinal barrier, with subsequent uptake of immunogenic peptides into the lamina propria that induce an adaptive proinflammatory response in individuals who carry the HLA-DQ2 and DQ8 genes [4]. Impairment of mucosal integrity in active CD is not restricted to the small intestine and has been detected in other columnar digestive epithelia, such as the gastric, intestinal, and colonic mucosa [5, 6]. It is unclear whether patients with active CD also present alterations in the esophageal mucosa.

Reflux symptoms (RS) can be a manifestation of gastroesophageal reflux disease (GERD), as a consequence of stomach contents flowing into the esophagus and causing troublesome symptoms and/or complications [7]. Endoscopically, GERD is classified as erosive esophagitis or nonerosive reflux disease [8]. Up to 70% of patients with GERD have no macroscopic changes in the gastroesophageal junction and thus a number of functional and microscopic alterations, including loss of mucosal integrity, have been proposed to explain the presence of symptoms in these patients [9–12]. On the other hand, CD has a protean clinical presentation that includes a variety of gastrointestinal and extraintestinal symptoms [13]. RS are common in CD [14], affecting almost 70% of patients, with 30% of them considering symptoms to be moderate to severe [15]. Notably, most CD patients with reflux symptoms have the nonerosive phenotype, are refractory to proton pomp inhibitors (PPI), and show fast improvement after starting the gluten-free diet (GFD) [15, 16]. Furthermore, the majority of patients will not experience a relapse as long as they maintain strict adherence to the diet [6–12]. The association between CD and RS is reinforced by the fact that CD is incidentally diagnosed during the endoscopic evaluation for GERD [15–19]. The mechanisms underlying reflux symptoms in CD patients are unknown, but esophageal motor disturbances, delayed gastric empting, and confirmed GERD have been suggested [20, 21]. However, the integrity of the esophageal mucosa in CD patients has not been investigated. We hypothesized that gluten-induced dysfunction of the esophageal mucosa may be involved in the generation of reflux symptoms in CD. We therefore studied intercellular spaces and expression of TJ proteins in the lower esophageal mucosa of celiac patients with newly diagnosed CD. When possible, we explored other aspects of esophageal function using manometry and 24-hour pH/impedance tests. Celiac patients were clinically phenotyped and stratified into those with and without RS. Results were compared with those obtained from patients with classical GERD and individuals undergoing upper endoscopy for causes other than GERD or CD, but not reporting RS (controls).

## 2. Materials and Methods

### 2.1. Patients, Controls, and Study Design

This observational study was conducted at “Dr. Carlos Bonorino Udaondo” Gastroenterology Hospital between 2012 and 2013. The study was approved by the Local Research and Ethical Boards and all participants signed the approved written informed consent. Consecutive adult (16 years or older) patients with newly diagnosed CD attending the Small Bowel Diseases Clinic were enrolled at the time of diagnosis, while still on a regular gluten-containing diet. Patients with former diagnosis of CD or already on a GFD or taking medication interfering with the study, such as proton pump inhibitors (PPIs) 2 weeks prior to the endoscopic procedure, or in whom esophageal organic disease was diagnosed were excluded from the study. Patients who declined to or were unable to participate in the initial and follow-up evaluation were also excluded. Consecutive patients attending the endoscopy unit with classical gastroesophageal RS and negative anti-tissue transglutaminase a-tTG (GERD patients), as well as subjects undergoing upper endoscopy for other causes than CD or GERD without RS and negative a-tTG (controls), were also enrolled.

After consent, patients and controls were asked to complete self-administered questionnaires. All demographic and symptom data were collected prospectively at the initial clinic visit. Basic sociodemographic data included age, gender, ethnicity, lifestyle (tobacco and alcohol), and concomitant medication (Table 1). All CD patients underwent a standardized upper endoscopic procedure performed at Dr. C. B. Udaondo Hospital, and esophageal and duodenal biopsies were obtained. The endoscopic appearance of the gastroesophageal junction in patients with RS was graded according to the Hill score [22]. The presence of esophagitis was categorized according to Los Angeles classification [23]. Biopsies from the distal esophagus were obtained 2 cm above the z-line. A second endoscopy, which included biopsies of the distal esophagus, was offered to participants after one year on a gluten-free diet.

The diagnosis of CD was based on clinical, serological, and histological grounds [2]. The diagnosis required the presence of a characteristic CD enteropathy (Marsh III or greater [24]) in the duodenal biopsy and positive IgA a-tTG and/or IgG anti-deamidated gliadin peptide antibodies (a-DGP) and/or IgA antiendomysial antibodies (EmA). Four biopsies were obtained from the distal duodenum. Histological damage in the duodenal biopsy was graded using the Marsh modified classification and the most severe damage was reported [24].

### 2.2. Patient Group Allocation Based on Gastrointestinal Symptoms Rate Scale

After all data were collected, patients were allocated to one of the following four groups based on GSRS questionnaire: (1) CD with RS; (2) CD without RS; (3) non-CD patients with RS (GERD patients); and (4) non-CD without RS (controls).

The presence of RS was assessed by a subdimension in the Gastrointestinal Symptoms Rating Scale (GSRS) that focuses on heartburn and acid regurgitation symptoms and is scored on a 7-graded Likert scale [25]. The GSRS is a disease-specific instrument and has shown good reliability; it discriminates symptom severity and is useful for evaluating treatment outcomes. The GSRS scores range from 0 to 6 for heartburn and regurgitation, where 0 signifies no symptoms and 6 represents the highest severity. An average score of >2 for both items was considered indicative of moderate to severe RS [15].

### 2.3. Light and Electronic Microscopy of the Esophageal Mucosa

Samples were assessed by two investigators (YW, XH) with experience in pathology and a third investigator (EV) who independently assessed the histological damage and dilated intercellular spaces (DIS) scores by light microscopy (LM) and electron microscopy (EM). Based on orientation and presence of basal layer and subepithelial tissue, biopsies were evaluable in 18 CD patients, 6 controls, and 6 GERD patients (Supplementary Table 1 in the Supplementary Material available online at http://dx.doi.org/10.1155/2016/1980686). The tissue was used for the morphological analysis using the protocol and score previously reported [26]. After conventional transmission electron microscopic processing of samples, semithin sections (0.5 *μ*m) were cut from three blocks of each case and stained with 1% toluidine blue. All sections were examined sequentially under the light microscope. For the semiquantification, degree of the dilated intercellular space in the basal layer and lower prickle layer of the esophageal epithelium was scored from 0 to 4 (Figure 1 from Supplementary Table 2). Areas with intercellular space dilation were identified when the diameter of most intercellular spaces at the area was greater than 2.5 *μ*m [26]. A final score (range 0–12) was calculated after adding the individual scores from a set of 3 blocks.

For EM, samples were prefixed for 2 h in 0.1 M sodium cacodylate buffer (pH 7.4) and glutaraldehyde. Subsequently, samples were transferred to 0.2 M sodium cacodylate buffer (pH 7.4), incubated for 12 h at 4°C and transferred after fixation in osmium tetroxide for 1 h to obtain block of resine. From the semithin sections, ultrathin sections (70–80 nm) were cut, placed on 100-mesh copper grids, and stained with uranyl acetate and lead citrate. All sections were studied completely with a transmission electron microscope (Jeol 1200EX Biosystems, Japan). For quantification of the EM, three electron microscopic photos (same size and same magnification, ×3000) of epithelial cells from the basal and lower prickle layers were chosen from each case. They were analyzed using Photoshop. Intercellular space was delineated using density slicing on grey scale images. The area of intercellular space was measured and expressed as percentage of the total area (pixel numbers). The LM and EM assessment was performed by a single expert in a blinded fashion.

### 2.4. Real-Time Quantitative Reverse Transcription PCR (Real-Time qRT-PCR) for Tight Junction mRNA Expression

Total RNA was extracted from esophageal biopsy sections using an RNA easy mini kit (Qiagen Sciences, Ontario, Canada) and treated with RNAse-free DNase set (Qiagen Sciences, Ontario, Canada) to remove contaminating DNA (cDNA). cDNA synthesis was performed using an oligo-dT primer and M-MLV reverse transcriptase (Invitrogen, Ontario, Canada). The cDNA samples were used to measure CLDN-2, CLDN-3, and ZO-1 by real-time quantitative PCR using SsoFast*™* EvaGreen Supermix (Bio-rad, Ontario, Canada) and iQ5 system (Bio-rad, Ontario, Canada). The primers are described in Supplementary Table 3; GAPDH gene was used as housekeeping gene. The PCR was performed at 95°C for 30 s, followed by 30 cycles (ZO-1 and GAPDH) or 36 cycles (CLDN-2) or 38 cycles (CLDN-3) at 95°C for 6 s, and 60°C for 10 s; then a melt curve analysis from 55–95°C (in 0.5°C increments) was conducted. The PCR efficiencies were measured for each primer pair. The analysis of relative gene expression levels was completed by the 2-ΔΔCT method. Primer sequences and product sizes used for determination of the mRNA expression of TJ proteins are shown in Supplementary Table 3.

### 2.5. Double-Immunofluorescence for Anti-Tissue Transglutaminase 2 (Anti-TG2) IgA Deposits

Frozen esophageal biopsy sections (4 *µ*m) were fixed with cold acetone and then incubated for 1 h at room temperature with a blocking solution consisting of 5.0% normal rabbit serum in 0.1 M PBS, pH 7.4, and then overnight at 4°C with a monoclonal mouse primary antibody against human TG2 (CUB 7402) (1 : 40, Abcam, Ontario, Canada). The second day, the sections were incubated for 45 min in the dark at room temperature with a mixture of fluorescein isothiocyanate- (FITC-) labeled polyclonal rabbit antibody against human IgA (1 : 50, Dako, Ontario, Canada) to detect IgA deposits (in green) and Alexa Fluor® 594-labeled polyclonal rabbit secondary antibody against mouse (1 : 200, Invitrogen, Ontario, Canada) to detect TG2 (in red). The sections were mounted with antifade mounting medium with DAPI. Colocalization of anti-TG2 IgA deposits (in yellow) was visualized using Image-Pro Plus version 6.3. Negative controls were performed with the absence of primary antibodies.

### 2.6. Esophageal Manometry and 24-Hour pH/Impedance Monitoring

Manometry and 24-hour pH/impedance tests were performed as a pilot study in a subset of patients, 12 CD (9 with reflux symptoms) and 18 GERD; none of the patients was on antisecretory medication. We used a water-perfused manometry system (Arndorfer Inc., Greendale, Wisconsin, US) with 8-lumen silicone rubber extrusion catheter with a 6 cm sleeve sensor and 8 side-hole recording sites. Localization, pressure, length, and % relaxation of lower esophageal sphincter (LES) and upper esophageal sphincter (UES) were measured at end expiration, during a 5-minute baseline period.

After manometry, 24-hour pH/impedance test was performed using a combined pH-MII flexible probe (AccuTrac pH-Z; Sierra Scientific Instruments, Canada). The data obtained was analyzed by AccuView*™* software. Total number of reflux episodes, percent of total time with pH < 4, type of reflux (acidic, weakly acidic, or alkaline), and DeMeester score were reported.

### 2.7. Statistical Analysis

The statistical analyses were carried out using IBM SPSS (IBM SPSS Statistics Version 22, Chicago, IL, USA). Continuous variables and scores were expressed as means and standard deviations (SD) or median and interquartile range (IQR) as appropriate. Categorical variables were expressed as percentages. Comparison of continuous and categorical variables between groups was performed using ANOVA, Kruskal-Wallis, Student's *t*-test, Mann-Whitney *U* test, and *χ* ^2^ test, as appropriate. When ANOVA was performed, a Bonferroni correction was applied. *p* < 0.05 was considered statistically significant.

## 3. Results

### 3.1. Characteristics of the Study Population

Overall, 55 subjects were enrolled in the study, including 25 CD patients, 19 GERD patients, and 11 controls. All three groups were comparable in demographic characteristics as shown in Table 1. From a clinical point of view, body weight, body mass index, and those risk factors for GERD (smoking status, hiatal hernia and its size, EE, appearance of the gastroesophageal junction, and the endoscopic grading of flap valve) were comparable between CD and GERD patients (Table 1). All CD patients, but no GERD patients or control, had positive CD specific serology. Endoscopy showed no major alterations such as Barrett's esophagus or cancer in esophageal junction in any of the subjects. According to the reflux domain of the GSRS questionnaire, 16 out of 25 CD patients (64%) had scores categorized as moderate to severe symptoms (>2 points). As expected, all patients with RS had abnormal GSRS scores while controls had scores of 0. A summary of tests performed in all groups and reasons for no test availability in individual patients is shown in Supplementary Table 1.

### 3.2. Dilated Intercellular Spaces (DIS) Assessment

**Figure 1:**
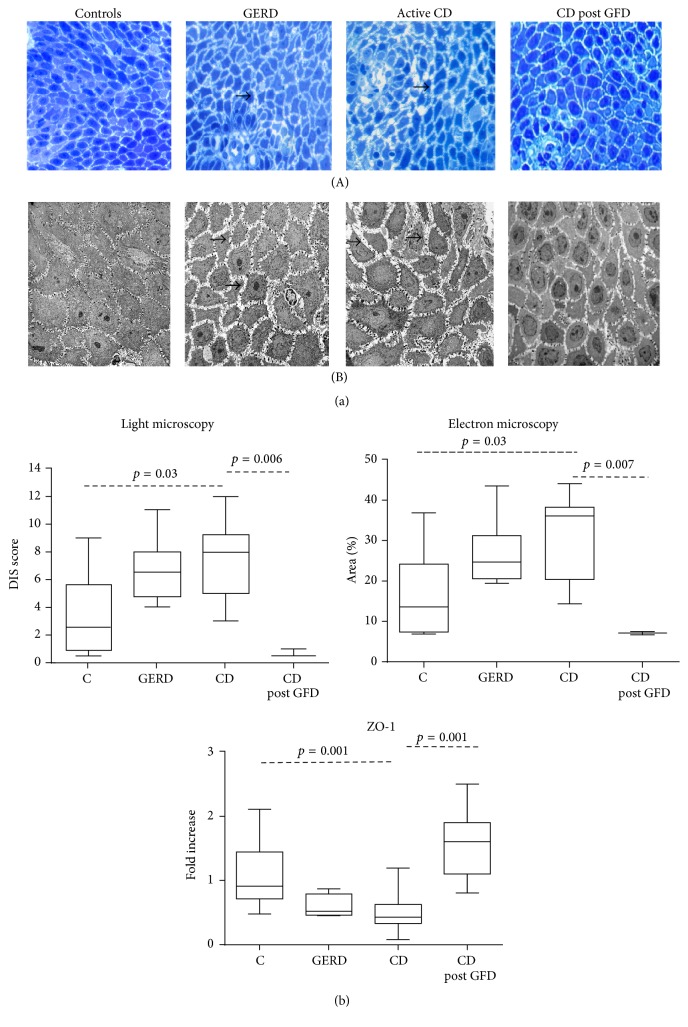
(a) Examples of intracellular spaces in the esophageal mucosa of controls (C), GERD patient, active CD patient before gluten free diet (CD), and CD patient after one year of gluten-free diet (CD post GFD) assessed by light microscopy (A) and electron microscopy (B). Black arrows indicate dilated intracellular spaces (DIS). (b) Group DIS scores assessed by light microscopy, electron microscopy, and mRNA expression for zonula occludens-1 (ZO-1).

LM and EM assessment was performed in high quality biopsies of 18 CD patients (9 with RS and 9 without RS), in 6 GERD patients and 10 controls (Figure 1). The biopsies were not evaluated when the experts considered the tissues samples were not appropriate based on incorrect orientation or insufficient basal cell layer (Supplementary Table 1). CD samples were pooled as no differences were found between CD with or without RS (LM: median [IQR]: 8.0 [4.0–9.2] and 8.0 [6.0–10.5]; EM: 34.9 [17.7–38.4] and 36.1 [34.0–37.4], resp., all *p* = NS).

There were significant differences in DIS scores assessed by LM and EM between groups (LM *F* = 3.85, df = 2, and *p* = 0.03; EM *F* = 3.87, df = 2, and *p* = 0.03). LM analysis showed that controls had DIS within the normal range and the overall score was lower than that in CD patients (Figure 1(b)). No differences in DIS were observed between CD and GERD patients. EM assessment showed higher scores DIS in CD patients compared to controls (*p* = 0.02). As expected, GERD patients also had higher DIS scores compared with controls (*p* < 0.001). The correlation between both methods (LM and EM) was excellent (Spearman's rank correlation *r* = 0.98).

### 3.3. Extraction of mRNA and Quantitative Reverse Transcription-Polymerase Chain Reaction (RT-PCR) of Tight Junction-Related Genes

We measured mRNA expression for ZO-1, CLDN-2, and CLDN-3 in 31 high quality biopsies with preserved RNA: 18 CD patients (10 with and 8 without RS), 4 GERD patients, and 9 controls. Details on samples included in the analysis and reasons for exclusion are provided in Supplementary Table 1. There was no difference in CLDN-2 and CLDN-3 mRNA expression between CD patients, GERD, and controls. In contrast, there was a significant difference in the mRNA expression of ZO-1 between groups (*F* = 8.02; df = 2; *p* = 0.002). The mRNA expression of ZO-1 was lower in CD and GERD patients compared to controls (CD versus controls = 0.001; 95% CI = 0.21–0.97). ZO-1 expression was similar in CD patients with and without RS (0.5 [0.5–0.8] and 0.9 [0.7–1.4], resp.). The expression of ZO-1 increased after one year of GFD in 8 CD patients irrespective of the presence of RS (*p* = 0.001) (Figure 1).

### 3.4. Assessment of IgA-TG2 Deposits in the Esophageal Mucosa

**Figure 2:**
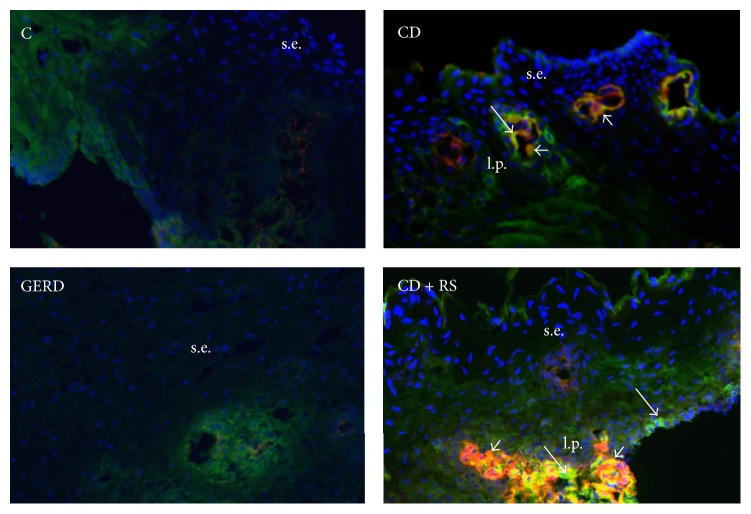
Example of immunofluorescent staining in the esophageal mucosa of a healthy control (C), a CD patient without RS (CD), a GERD patient, and a CD patient with reflux symptoms at the time of diagnosis (CD + RS) (magnification 20x). tTG2 (red); IgA deposits (bright green, perivascular, long arrows); IgA-TG2 deposits (orange/yellow, short arrows); s.e.: squamous epithelium; l.p. = lamina propria. Dull green background in C and GERD due to autofluorescence and nonspecific staining.

IgA-TG2 deposits in the esophageal lamina propria were assessed in the majority of patients with CD and GERD and in controls (Supplementary Table 1). Deposits of IgA-TG2 in lamina propria were observed in CD patients with and without RS but not in GERD patients or in controls. There was some degree of autofluorescence and background stain in many preparations including negative controls, and this was not considered an IgA deposit. IgA specific stain was bright green and usually in lamina propria and around blood vessels. This was observed in samples from CD patients but not in controls or GERD. Examples of immunostaining are shown in Figure 2.

### 3.5. Esophageal Manometry and 24-Hour pH-Impedance Tests

In a pilot study, we assessed 36 subjects by esophageal manometry (18 CD and 18 GERD patients). Thirteen out of 18 CD patients had moderate to severe reflux symptoms, while 5 were asymptomatic. The LES pressure of CD patients was higher than in GERD patients (Supplementary Table 4).

Thirty subjects were assessed by 24-hour pH/impedance test, including 12 CD patients (9 with RS) and 18 GERD patients (Supplementary Table 4). Patients with CD had more frequent nonacid reflux and less acid reflux, while no differences in total number of reflux episodes were observed between CD and GERD patients.

## 4. Discussion

In the present study we investigated putative pathophysiological factors involved in the genesis of RS, which are frequently reported by patients with active CD. The study involved patients without and with RS, classified as moderate to severe at the time of diagnosis. We also tested asymptomatic subjects (controls) and patients with GERD without CD. Similarly to our previous study [15], the majority of CD patients with RS became asymptomatic after starting the GFD, without requirement of additional therapy with PPIs. The prevalence of erosive esophagitis detected in the cohort of CD patients was lower than that expected for GERD patients with moderate to severe RS [15]. This observation contrasts with two previous studies, which have shown increased prevalence of endoscopic esophagitis in adult CD patients compared with nonceliac controls [16, 17]. The low prevalence of erosive esophagitis found in our study and the reversion of reflux symptoms by the GFD suggest that pathophysiological mechanisms determining the presence of RS in CD patients are different from those in patients with conventional GERD.

The most important finding in this study is related to mucosal structure changes in the distal esophagus of active CD patients, a factor that has not been investigated previously. The concept that loss of mucosal integrity can be relevant for the development of RS has been explored in patients with GERD and eosinophilic esophagitis by measuring DIS (using LM and EM) as well as the expression of TJ proteins and by assessing transepithelial electric resistance and permeability [10, 12, 27, 28]. Furthermore, the reversibility of DIS induced by the administration of PPIs has also been shown in patients with GERD [29]. In this study, we found increased/altered DIS in CD patients compared to controls and comparable to conventional GERD patients. The magnitude of DIS was similar in CD patients with and without RS. Mucosal integrity was restored to values similar to those of controls after one year of treatment with the GFD, a finding which correlated with the remission of symptoms, without PPI therapy. These observations imply a role for impaired esophageal mucosal integrity in active CD and could explain the presence of RS in, at least, a subgroup of patients. The reversion of symptoms after the GFD can be attributed to the restoration of mucosal integrity, since patients did not undergo any specific therapeutic interventions for RS, other than the dietary modification.

Another novel aspect investigated in this study was the measurement of gene expression for TJ proteins in esophageal mucosa from active CD patients. Although our study did not find differences in the expression of CLDN-2 and CLDN-3 between CD and controls, the expression of ZO-1 was lower in both CD (with or without RS) and GERD patients compared to controls. Claudins, occludin, and ZO-1 are all considered key elements of the TJ complex and their expression and localization dictate TJ competency, particularly in the intestine [30–33]. Although less is known about TJs and their relation to paracellular permeability function in the esophageal mucosa, some studies have suggested an altered expression of TJ proteins in GERD [10, 28]. Our global analysis of differential expression of CLDN-2, CLDN-3, and ZO-1 in these well-defined populations suggests that the underlying mechanisms for RS and mucosal changes in patients with active CD are different from those in GERD patients. There is evidence that impaired barrier function of esophageal mucosa is a “hallmark” of GERD [28]. Furthermore,* in vitro* studies have demonstrated that acid as well as bile salts in the refluxate influenced paracellular permeability in the esophageal epithelium [34]. On the other hand, impaired epithelial integrity in the duodenal mucosa has been described in CD even during early stages [35] and gliadin has been suggested to regulate the expression of CLDN-3, CLDN-4, and, in particular, ZO-1 in celiac patients [31, 36]. However, the role of esophageal TJ in CD patients in relation to RS is unclear. The altered gene expression for ZO-1 detected in this study could lead to esophageal permeability alterations and constitute a potential mechanism that explains symptom generation. Interestingly, the expression of ZO-1 was increased in CD patients with and without RS and normalized one year after the GFD, supporting the hypothesis that changes in some TJ proteins are related to the celiac condition rather than being a mere consequence of acid reflux. Since TJ mRNA expression was altered in CD patients independently of the presence of RS, it is possible that other mechanisms along with TJ changes contribute to RS development. Moreover, the assessment of isolated mRNA may not reflect the comprehensive role of ZO-1 or other TJ proteins, and thus these results will need to be confirmed at the protein level in the future.

Distal esophageal function was evaluated by manometry and 24-h pH-impedance tests in a subgroup of patients that consented to these tests. While GERD patients exhibited mildly hypotensive LES, CD patients had normal LES tone. The 24-h pH/impedance test then showed abnormal gastroesophageal reflux in most CD patients, irrespective of the presence of RS. Up to now, only two studies [16, 20] performed in small cohorts of patients have demonstrated pathological acid reflux by pH-metry in patients with active CD. In our study, reflux was acid in all patients with GERD, while most CD patients displayed mainly weakly acidic or nonacid reflux. No differences were observed in total number of reflux episodes between CD and GERD patients; however, shorter episodes of reflux were observed in CD patients. In addition to the effect of acidic reflux, duodenal reflux components, such as bile salts and trypsin, have the potential to disrupt esophageal barrier function, partly by modulating TJ proteins [34], and this could be a possible mechanism for esophageal mucosal integrity disruption in CD patients. However, these preliminary functional observations need to be confirmed in larger cohorts of patients.

Finally, we explored the possibility that autoimmunity could play a role in the presence of RS in patients with active CD. We based our hypothesis on the fact that CD is an autoimmune disorder with systemic presentation (digestive and extradigestive) and on the recognition of transglutaminase 2 as the autoantigen in CD. In fact, tissue deposits of IgA antibodies to TG have been found in the intestinal mucosa [37], skeletal bones [38, 39], thyroid tissue [40], liver [41], and placenta [42]. Moreover, IgA antibody granular deposits against TG3 were suggested to have specific diagnostic value in dermatitis herpetiformis [43]. Here we show IgA TG2 deposits in the esophageal mucosa of all CD patients in whom this could be measured, but not in controls or GERD patients. Although the presence of TG2 in the esophagus does not preclude a role in the development of RS in patients with active CD, it contributes to the notion of generalized autoimmunity in CD that could be detected beyond the small bowel. Although TG2 after GFD was not assessed in our cohort of patients, this analysis is encouraged for future studies.

A major limitation of our study is the disparity in numbers of subjects and samples included in individual tests due to logistical problems such as obtaining consent to perform invasive studies in controls.

In conclusion, our study explored possible mechanisms underlying the expression of RS in a group of patients with active CD assessed at the time of diagnosis and after one year on GFD. We found that DIS is a common finding in the distal esophageal mucosa of patients with active CD. This abnormality in esophageal mucosal integrity seems to be part of a generalized permeability alteration affecting the digestive tract of CD patients. Although the morphological characteristics of DIS in CD seem to be similar to those occurring in patients with conventional GERD, there are some distinctions in at least some of the TJs involved, with ZO-1 potentially playing a role in esophageal mucosal integrity in CD. The changes also appear to be gluten-dependent as the alterations revert with the GFD, along with symptomatic improvement. Altogether, our results suggest that the underlying mechanism for RS generation in CD is different from that in patients with conventional GERD and is a gluten-driven phenomenon. We believe that these novel findings provide new insight into the pathophysiology of RS in CD patients and will stimulate clinical research in this area.

## Supplementary Material

Additional data related to the characteristics of study population, intercellular spaces scores, cDNA primers, and Ph-Impedance and manometry described in methods and results sections will be available online as supplementary materials.

## Figures and Tables

**Table 1:** Characteristics of the study population.

Subjects	CD *N* = 25	GERD *N* = 19	Asymptomatic controls, *N* = 11	*p* value^*∗∗*^
Age, median (range)	28 (18–73)	44 (24–68)	36 (28–62)	0.04
Female, number (%)	17 (68)	5 (45)	3 (60)	0.41
BMI, mean (SD)	23 (3)	24 (2)	25 (2)	0.23
a-tTG IgA, mean (SD)	189 (125)	2 (0)	2 (1)	<0.001
a-DGP IgG, mean (SD)	88 (116)	3 (2)	0 (1)	<0.001
a-DGP IgA, mean (SD)	129 (49)	7 (3)	1 (1)	<0.001
Smokers, number (%)	2 (8)	1 (2)	3 (27)	0.15
Alcohol (units/week), mean (SD)	1 (1.9)	1.2 (1.4)	3.8 (4.7)	0.18
Hiatal hernia size (cm), mean (SD)	1 (1)	1 (1)		0.86
Esophagitis, number (%)	3 (12)	2 (11)		0.99
Endoscopic grading flap valve (Abnormal Hill), number (%)	8 (32)	7 (37)		0.74
Intestinal mucosal damage, number				
Marsh IIIa	1			
Marsh IIIb	6			
Marsh IIIc	18			

^*∗∗*^CD versus controls; Mann-Whitney *U* test.

## Abbreviations

CD: Celiac disease
RS: Reflux symptoms
GERD: Gastroesophageal reflux disease
C: Controls
IS: Intercellular spaces
NERD: Nonerosive reflux disease
EE: Erosive esophagitis
DIS: Dilated intercellular spaces
TJ: Tight junction
GFD: Gluten-free diet
CLDN-2: Claudin 2
CLDN-3: Claudin 3
a-tTG: Anti-tissue transglutaminase antibody.

## Key Messages

Consider the following. (i) Reflux symptoms are common in patients with active celiac disease (CD) but the mechanism for symptom generation is poorly understood, (ii) CD patients become asymptomatic after starting the gluten-free diet, without requirement of additional therapy with proton pump inhibitors, and (iii) patients with active CD have altered esophageal mucosal integrity, evidenced by dilated intracellular spaces and altered of ZO-1 mRNA expression compared to controls.
