# Supplementary material for: Altered Esophageal Mucosal Structure in Patients with Celiac Disease
Source: Can J Gastroenterol Hepatol. 2016 Feb 29;2016:1980686. doi: 10.1155/2016/1980686 (PMC4904646; doi:10.1155/2016/1980686)
Supplement: Supplementary file 1 — Additional data related to the characteristics of study population, intercellular spaces scores, cDNA primers, and Ph-Impedance and manometry described in methods and results sections will be available online as supplementary materials. [file 1980686.f1.doc]

**Supplementary Table 1**: Details of tests available and reasons for not available

| **Test/groups** | **Available** | **Not available** | | |
| --- | --- | --- | --- | --- |
|  | **Test available (n)** | **Biopsy insufficient /not evaluable (n)** | **Patient refused (n)** | **Lost to follow-up (n)** |
| **GSRS**  CD+RS+  CD+RS-  GERD  AC | 16  9  19  11 | -  -  -  - | -  -  -  - | -  -  -  - |
| **DIS- LM**  CD+RS+  CD+RS-  GERD  AC | 9  9  6  10 | 7  -  13  1 | -  -  -  - | -  -  -  - |
| **DIS- EM**  CD+RS+  CD+RS-  GERD  AC | 9  9  6  10 | 7  -  13  1 | -  -  -  - | -  -  -  - |
| **CL2**  CD+RS+  CD+RS-  GERD  AC | 4  4  4  8 | 12  5  15 | -  -  - | -  -  - |
| **CL3**  CD+RS+  CD+RS-  GERD  AC | 10  8  4  9 | 6  1  15  2 | -  -  -  - | -  -  -  - |
| **ZO1 Baseline**  CD+RS+  CD+RS-  GERD  AC | 10  8  4  9 | 6  1  15  2 | -  -  -  - | -  -  -  - |
| **ZO1 Post GFD**  CD+RS+  CD+RS- | 4  2 | -  - | -  - | 12  14 |
| **Manometry**  CD+RS+  CD+RS-  GERD  AC | 13  5  18  3 | -  -  -  - | 3  4  1  8 | -  -  -  - |
| **PH-Impedance**  CD+RS+  CD+RS-  GERD  AC | 9  3  18  3 | -  -  -  - | 7  6  1  8 | -  -  -  - |
| **TTG-2**  CD+RS+  CD+RS-  GERD  AC | 14  8  5  9 | 2  -  14  2 | -  -  -  - | -  -  -  - |

**Supplementary Table 2**: Semi quantitative score for assessment of intercellular space by light microscopy.

| Score | Description | |
| --- | --- | --- |
| 0 | There is no or less than ¼ area with dilated intercellular space |  |
| 1 | There is < ¼ area with dilated intercellular space |  |
| 2 | There is > ¼ but <½ area with dilated intercellular space |  |
| 3 | There is > ½ but < ¾ area with dilated intercellular space |  |
| 4 | There is > ¾ area with dilated intercellular space |  |


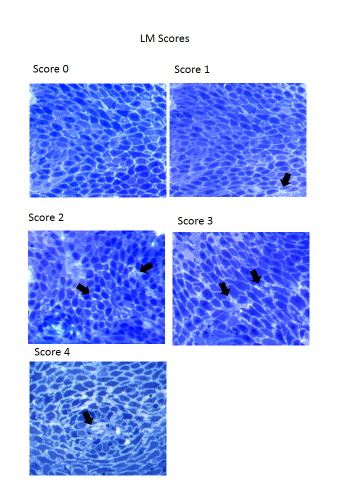


**Supplementary Table 3**: Primer sequences and product sizes used for determination of the expression of TJ proteins.

| **Table 1 Primer sequences and product sizes** | | | |
| --- | --- | --- | --- |
| Primer | Forward sequence 5'-3' | reverse sequence 5'-3' | Product size(bp) |
| Claudin-2 | TCTCTTGGCCTCCAACTTGTGGG | GCACTGGATGTCACCATCATGGC | 259 |
| Claudin-3 | CTGCTCTGCTGCTCGTGTCC | TTAGACGTAGTCCTTGCGGTCGTAG | 128 |
| ZO-1 | TCTGATCATTCCAGGCACTCGC | CCACATCTGGTTGCCAACTTGG | 225 |
| GAPDH | GAAGATGGTGATGGGATTTC | GAAGGTGAAGGTCGGAGT | 225 |

**Supplementary table 4**: 24-h ambulatory pH/Impedance and manometry assessment in CD and GERD patients

|  | **GERD^#^ (n=18)** | **CD (n=12)** | **P value^**^** |
| --- | --- | --- | --- |
| **24-h pH-Impedance test** |  |  |  |
| Total episodes of acid reflux | 26.5 (8.7-35.0) | 7.5 (2.2-17.0) | 0.07 |
| Total episodes of weakly acidic reflux | 6.5 (4.0-15.7) | 16.0 (7.7-23.5) | 0.02 |
| Total episodes of non-acid reflux | 0 (0.0-0) | 15.0 (2.5-25.0) | <0.001 |
| Total episodes | 35.5 (18.7-51.2) | 44.5 (22.5-70.7) | 0.37 |
|  |  |  |  |
| **DeMeester Score** |  |  |  |
| Total time with pH ≤4 (min) | 83.0 (38.0-192.2) | 0.0 (0.0-2.0) | <0.001 |
| Total time with pH ≤4 up-right (min) | 44.0 (20.7-114.7) | 0.2 (2.8-2.6) | <0.001 |
| Total time with pH ≤4 supine (min) | 35.5 (9.25-67.5) | 0.0 (0.0-0.9) | <0.001 |
| Longest episode duration (min) | 13.5 (5.75-35.2) | 0.0 (0.0-0.0) | <0.001 |
| Total number reflux episodes >5 min | 3.5 (0.7-9.5) | 0.0 (0.0-0.7) | 0.002 |
| Total DeMeester Score | 31.0 (21.5-69.7) | 2.5 (0.9-7.2) | <0.001 |
| Abnormal pH-impedance test, n (%) | 16 (88) | 10 (83) | 0.99 |
|  |  |  |  |
| **Manometry test** | **GERD^#^ (n=18)** | **CD (n=18)** | **P value^**^** |
| LES length (cm) * | 3.0 (2.0-3.1) | 2 (2.0) | 0.07 |
| LES pressure * (mm Hg) | 9.4 (7.5-11.4) | 18.6 (14.0-27.0) | <0.001 |
| UES length (cm) * | 2.0 (2.0-3.0) | 2.5 (2.0-3.0) | 0.44 |
| LES hypotensive, n (%) | 13 (72) | 1 (5) | <0.001 |

*Median (IQR), ** Mann-Whitney U test, ^#^ Chi^2^ test

^#^ The definition of GERD is based in GSRS score >2 (non-celiac population with moderate to severe reflux symptoms)

**
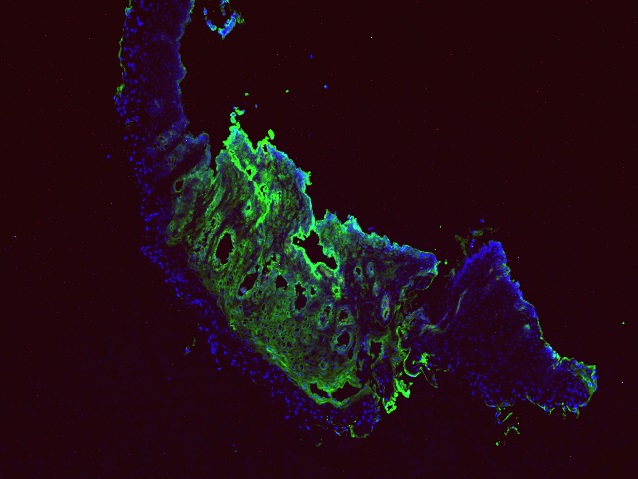

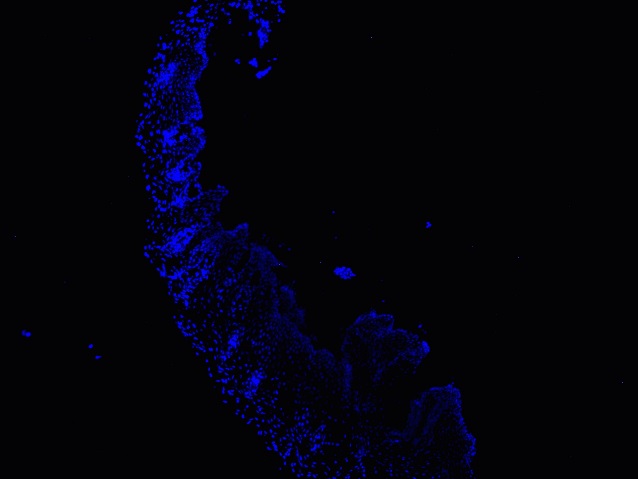
**

**Supplementary figure 1**: **A)** Example of immunofluorescent staining showing IgA (bright green) deposits in the esophageal mucosa of a CD patient without RS (CD) (4x). **B)** Negative control without the use of primary antibody for IgA.

**A**

**B**
